# Social participation in evidence: the memorable 17^th^ National Health Conference

**DOI:** 10.1590/0102-311XEN154023

**Published:** 2023-09-18

**Authors:** Claudia Travassos, Luciana Dias de Lima

**Affiliations:** 1 Fundação Oswaldo Cruz, Rio de Janeiro, Brasil.; 2 Escola Nacional de Saúde Pública Sergio Arouca, Fundação Oswaldo Cruz, Rio de Janeiro, Brasil.

We live in times when communication happens quickly, almost simultaneously to the episodes it describes. Events of high social and political impact, such as the 17^th^ National Health Conference (CNS), are objects of dissemination and analysis that happen nearly in real time. Less than a month before the ending of the Conference, much has been written, broadcast, commented on and debated ^1^ ^,^ ^2^ ^,^ ^3^. These analyses express a great consensus about the 17^th^ CNS’s historical milestone: the exuberant presence of the Brazilian people’s diversity.

A sense of magic and joy embraced those present, who, sharing a common space, perceived that they were united by the solidarity objective of “guaranteeing rights, defending the SUS, life, and democracy” and building a new tomorrow for healthcare. More than 6,000 people participated, including guests and delegates nominated by the States and the Federal District Conferences and by the 99 free conferences, who for the first time were able to elect representatives and submit proposals, significantly expanding social participation and the topics under debate.

The 17^th^ CNS took place in a setting of celebration of the victory of democracy and the urgency of (re)building the country. The far-right candidate, defeated in the 2022 elections, sought re-election by manipulating State institutions and resources in his favor. Such candidate had become popular by following the international far right’s strategies and methods, marked by denialism, spread of a culture centered on hatred and death, compulsive use of misinformation and coup attempts, such as the one that happened on January 8, 2023. Under his misgovern, Brazil experienced the COVID-19 pandemic, becoming one of the countries with the highest mortality rate for the infection. 

In the rooms of the Brazilian International Convention Center, where the 17^th^ CNS was held, people of various ages spoke in multiple accents, coming from all places and spaces of Brazil; different skin-colors mingled with the LGBTQIA+ pride flags’ rainbows and with the colorful and creative headdresses of the Indigenous peoples that marked their presence, along with the largest delegation of people with disabilities ever present in a National Health Conference. The plurality of bodies and voices was present in corridors, occupied rooms, spread throughout themes and intensified debates. Diversity expressed by the innumerable social collectives and groups, all carrying their own demands and particular manifestations, expressing the strength of the social movement in health, in its capacity for mobilization and participation. Those who attended the Conference faced the amplified image of the plural and democratic character of President Lula’s inauguration and of a country proud of its plurality. 

Definitely, the 17^th^ CNS’ protagonist was the social movement. But the academy was also present and shared the activities with other participants, contributing to the self-managed activities and to the working groups’ debates. Its participation in the process of building the 17^th^ CNS stands out in the articulation with the social movement for the organization of free conferences. This process began with the holding of the Free, Democratic and Popular Conference on August 5, 2022, organized by the *Frente pela Vida*, an entity created during the COVID-19 pandemic by public health scientific organizations. The Free, Democratic and Popular Conference was built by holding several free conferences, stimulating this participation modality. 

The *Frente pela Vida* entity opposed the denialism and death policies conducted by the past government, through actions that articulated and helped (re)organizing the social movement in defense of life, the SUS and the democracy. It contributed with guidelines and proposals aimed at the universal, public and egalitarian SUS. It is an innovative movement that seeks to adopt new arrangements in academy’s relationship with the social movement for the joint construction of proposals and of the democratic exercise. Aiming toward innovation within the social movement, the *Mapa Colaborativo dos Movimentos Sociais em Saúde* (Collaborative Map of Social Movements in Health) was launched by the National Health Council at the Conference ^4^. At the opening ceremony, the Minister of Health announced the National Health Council’s approval of *Resolution n. 714*, of July 2, 2023 ^5^, which deals with the creation of Local Health Councils in the basic health units (UBS), an act of strengthening social control in the SUS.

The large social participation in the 17^th^ CNS was followed by a significant number of submissions forwarded by the movements of “minorities” (race, gender, ethnicity, people with disabilities, among others) and associated with specific causes (palliative care, victims and relatives of victims of COVID-19, rare diseases). Such participation expanded the themes included in the guidelines and proposals that composed the Consolidated National Report submitted to the Conference’s deliberation. In this context, those proposals directed to specific demands were more prevalent than those related to structural issues such as financing, assistance model and deprivatization strategies. The social health movement showed its progressive political character, with conservative guidelines and proposals being systematically defeated in the Conference’s Final Full Court.

The same result was achieved in the *Resolution n. 715*, of July 20, 2023 ^6^, which provided strategic guidelines for the Multiannual Plan and the National Health Plan of 2024-2027 from the 17^th^ CNS, on the priorities approved by the National Health Council. As a result of this process it is possible to identify, on the one hand, advances for the guarantee of rights; on the other hand, there is an evident need for demands for greater inclusion to be accompanied by policies that guarantee an universal, integral, egalitarian and high-quality healthcare system.

The 17^th^ CNS took place at a moment in which conservative and privatism-led forces of the Brazilian National Congress were pushing for the replacement of Minister of Health Nísia Trindade Lima. Acclaimed by the CNS, the Minister of Health identified herself as the “Minister of SUS”, legitimized in the speech delivered by President Lula at the Conference’s closing ceremony, which ratified the importance of the struggle against fascism and in favor of social participation and the SUS. However, the advances in guaranteeing rights present in the CNS’ recommendations were repudiated by conservative groups in the Congress, especially by the evangelical group, which tried to prevent any development in this field ^7^.

“*We must learn to live with adversity, with the collective*” ^8^ (p. 42, free translation). These words were spoken by Sergio Arouca in his presentation at the VIII National Health Conference in 1986. If at that moment we celebrated the participation of users among about 4,000 delegates, 37 years later we celebrate the environment of unity in the diversity provided by the SUS and the expansion and diversification of the social movement in health.

Long live democracy! Long live the SUS!
